# Basis for a Qualitative Research Project: Introduction to Qualitative Research

**DOI:** 10.17533/udea.iee.v44n1e16

**Published:** 2026-03-31

**Authors:** Carmen de la Cuesta Benjumea

**Affiliations:** 1 Nurse, Ph.D. Honorary Collaborating Professor, Department of Health Psychology. University of Alicante, San Vicente del Raspeig, Spain. Alicante Institute for Health and Biomedical Research (ISABIAL), Alicante, Spain. Email: ccuesta@ua.es https://orcid.org/0000-0003-2160-392X Universidad de Alicante Department of Health Psychology University of Alicante Alicante Institute for Health and Biomedical Research (ISABIAL) Alicante Spain ccuesta@ua.es

## 1. Essential characteristics of qualitative research

Qualitative research emerged as an alternative to positivist approaches to studying human experience and the ways in which people cope with problems.^1^ Von Wright points out that in the mid-19th century, the role of general laws of scientific explanation was questioned, and the problem arose as to whether theoretical construction was the same undertaking in the natural sciences as in the humanities and social sciences.^1^ As we know it today, qualitative research began in the 1960s with the development of symbolic interactionism and the grounded theory method. Since then, it has continued to evolve and innovate, while maintaining its essence ^2^ I discuss this in this introduction. The first thing to note, is that it is called qualitative because it reveals particularities of a given subject or phenomenon under study. The method provides information about the nature of the given subject; it tells us what it is made of. The purpose of qualitative studies is to achieve understanding in a Weberian sense, that is, to understand by putting oneself in the other's place and recognizing the intentionality of their action or behaviour; it is, therefore, an empathetic understanding. Qualitative research questions emphasize how a social experience is created and attributed meaning or significance, such as the experience of being the mother of a child with a disability or the experience of suffering from a serious illness. Through qualitative research, *meanings* and *processes* that people attribute to their experiences are revealed, placing them in *context.* ^3^

Let's pause for a moment to consider three key concepts: meaning, process, and context. Meaning refers to the interpretation that people give to situations, issues, rules, and behaviours; it provides us with a point of view. Meaning explains people's actions in a comprehensive, rather than causal, way. For example, expressing a patient's lack of adherence to treatment reflects the clinical point of view, while pointing out the limitations that treatment has on his social life indicates the meaning it holds for him. It is important here to distinguish meaning from opinions and judgments; meaning is not evaluative but rather expresses subjectivity. The process deals with the meaning attributed to the passage of time and its effects. It is therefore a temporality regardless of the time, day, or calendar month. Hence, it refers to the development of an experience. For example, a study that seeks to reveal the experiences of children receiving a liver transplant from the period prior to surgery, during surgery, and after surgery, ^4^ discusses this process.

When addressing context, people often think of the geographical and physical location, such as a specific city, country, or institution. Here, context is not so much the place itself but rather refers to the set of conditions and situations in which people act, and it is constructed in a discursive and symbolic way. These conditions and situations may include a country's public health policies, the characteristics of its human resources, the organization of its health system, or its level of development. For example, a review of the literature showed that the subject of family care has tended to be studied in developed countries that have conditions such as high employment rates, nuclear family structures, and support systems that people caring for a sick family member can turn to when the burden becomes excessive.^5^ It can also refer to places that have a situation such as remote, rural, and urban, in which case we speak of geographical context. Thus, the location of the study is not the object of study, although it conditions it due to the circumstances it brings together. 

Now that these three concepts have been clarified, we can move forward regarding the qualitative method for producing scientific knowledge and how it differs from the quantitative method. As they stem from different philosophical conceptions of science and paradigms, the methods are different but not contraries. This position respects diversity in the way we scientifically understand the world and places qualitative research on an equal footing with quantitative research. Neither is superior to the other; they are distinguished according to what we want to understand or know. Thus, as follows I will present the quantitative and qualitative methods, contrasting the objective, reasoning, analytic object approach, basic instrument and design of each, which are summarised in Table 1.

**Table 1:** Quantitative and qualitative research characteristics

Characteristic	Quantitative	Qualitative
Paradigm	Positivist	Interpretive/constructivist
Objective	Prediction and control	Understanding
Reasoning	Deductive	Inductive
Analytical object	Variable	Caso
Approach	Reductionist	Wholistic
Instrument	Survey/test	The researcher
Design	Predefined	Emerging

In general, quantitative research is associated with the modification and verification of theories, its objective being prediction and control. Qualitative research, on the other hand, is related to theory development and discovery, its objective is understanding in the Weberian sense mentioned above. Hence, in quantitative research, reasoning is deductive, i.e., there is a prior idea or concept to be tested or from which to start, while in qualitative research, reasoning is inductive, i.e., the idea or concept is formed based on the data generated.^6^ An example that well illustrates the difference between these types of reasoning can be found in Umberto Eco's book,^7^ where he recounts that when Marco Polo went to the island of Java, he said he saw a unicorn, but that it was ugly, with thick grey skin and a horn that was fat and short, like its legs. Marco Polo had seen a rhinoceros and not a unicorn, but he deductively applied a schema, that of a unicorn. Inductively, he would have given the animal a new name, thus “discovering” it.

For its part, the analytical object of quantitative research is the variable, while in qualitative research it is the case or phenomenon. Qualitative research studies all aspects comprehensively, without selecting variables in advance or fragmenting what is being studied, for the simple reason that it is yet to be discovered, so researchers view the phenomenon under study wholistically. ^8^ Therefore, the quantitative approach is reductionist, while the qualitative approach is comprehensive. The quantitative design is predetermined, the plan or project is carried out as proposed, and the qualitative design is emergent, with a flexible starting point that is developed by the researcher during the study itself. In this way, the researcher in qualitative studies is the instrument of the research, a crucial issue that deserves our attention.

### 1.1. The researcher's place in the research process

Unlike quantitative methods, where the researcher remains on the side-lines and separate from the research, in qualitative research the researcher is involved in the entire process and is therefore considered an instrument^2^. The researcher is the instrument in the design, in the generation of data, in its analysis, in the writing of the study, in maintaining ethical standards and in its quality. Let us look at each of these aspects. Regarding design, although there is a plan for carrying out the study, it is modified during the process to address limitations, fieldwork contingencies, and what the data reveals. The researcher is like a bricoleur, an artisan of his or her study. This is how the constructivist paradigm permeates the study; it is not given, but it is constructed in the act of researching.

The researcher is an instrument for generating data through the relationships s/he establishes with the study participants. If people do not trust him or her, s/he will clearly generate little data and of poor quality. Sudnow,^9^ in his seminal study, recounts that during fieldwork he made friends and this relationship allowed him to be in places reserved for staff and to learn things that would not be told to an outsider. The researcher is an instrument of data analysis through his/her interpretation of the data; his/ her erudition and sensitivity will be the tools that allow him or her to reveal meanings, processes, and contexts. When communicating what has been learned during the study, the researcher becomes a kind of intermediary between the point of view within the experience studied and the point of view outside it. S/he must produce a narrative that can convince and move readers, using metaphors, logic and persuasion to convey findings. 

Ethical issues are present throughout the research process, and it is the researcher's responsibility to maintain ethical standards. This refers to the axiological value of qualitative research. The production of qualitative knowledge requires an ethical position on the world and on oneself. Finally, the researcher's ability to reflect during the study on the effect his/her presence has on the participants and the effect the study has on them is an element of quality. ^10^ An example of this reflection can be found in the following account of a study on sudden infant death syndrome:

“I would like to describe what it was like for me to do this research. Although it took me a while to realize this, I eventually discovered that my research experience paralleled, to some degree, the parents' grief experience. Like the parents who were trying to rush their grief, I was trying to rush the research process. Like the parents who had to learn that grief takes its own time, I had to learn that this kind of research cannot be rushed. Eventually, I learned to let my understanding unfold naturally rather than trying to force it.” ^11^

Quality in qualitative research is not established by objectivity, but rather by the researcher's awareness of his/ her own subjectivity. This cannot be controlled, but one can be alert to it. It is not a problem to be subjective, but to believe oneself to be objective! All in all, who you are is important for the development of the study. In addition to having the necessary training to carry it out, various authors have referred to this point. It has been indicated that you must have certain qualities such as a liking for the humanities, be creative, have a sense of logic, be theoretically intelligent although sceptical about theory, possess a willingness to take risks and live with ambiguity; have an ability to solve problems in the field, a willingness to learn from people rather than study them, good conversational skills and even better listening ones.^12^ Do not be overwhelmed by this list, these qualities can be improved and acquired in the field. Schatzman and Strauss imply this when they state: “The researcher is a learner, has patience, is tolerant, and sympathetic. He [sic] wonders first and judges last; he [sic] appears to be this way; he *is* this way”. Indeed, when conducting qualitative research, a way of being develops, a researcher *self is* made. So, knowing the necessary qualities of a qualitative researcher, allows you to pay attention to them and refine them in practice.

## 2. Research deals with meanings and procedures

According to Charmaz, language shapes the structure of action. ^13^ Therefore, I present here a group of terms, inherited from positivism and present in qualitative literature, which have different meanings and procedures than those used in quantitative research. As in the early stages of language learning, I have chosen those that are relevant and frequently used. Knowing them will avoid misunderstandings and allow you to remain within the qualitative paradigm when conducting your study. Furthermore, knowing their appropriate meaning and how the procedures are carried out in the qualitative context, will let you to counter criticism and defend your work as a researcher. This is a first approximation; I will return to these terms and procedures in detail in subsequent articles.

### 2.1. Same name with different meanings

The terms explanation, sample, generalisation, design, data and results are, in my experience, the ones that cause the most confusion as, depending on whether they are used in a quantitative or qualitative context, they refer to different matters. The root of these differences lies in their paradigms. Let's look at each of these six terms. *Explanation.* When the term explanation is used in research, it is assumed that it must be causal, that is, it must report the reason for a phenomenon or event. However, in the context of qualitative research, the explanation is comprehensive; it must allow for an understanding of a phenomenon or experience in the Weberian sense. Thus, the quantitative explanation is the explanation that answers the ‘why?’ while the qualitative explanation answers the ‘how?’.

***Sample*.** In quantitative research, this refers to a part of a whole, usually a group or population. Qualitative research investigates phenomena rather than populations, so there is no sample in this sense, but rather an experience that is revealed in the study itself through sampling strategies. The equivalent here would be participants or informants; they do not constitute the sample as they do not represent a whole. Here there is an important nuance to bear in mind, that is: participants are participating in the research study because they participate of an experience or event, just for this reason are included in the study. Thus, during a study, the researcher must keep in mind the phenomenon being investigated, the *what* of the inquiry, avoiding distraction by the *who* in terms of external variables such as gender or level of education Therefore, in qualitative research, the sample consists of the study participants who have been chosen (sampling) for their experience and not for characteristics such as age and place of birth, unless the data show their relevance to the study. Although some publications already use the term participants to replace the positivist term sample, there are still documents, such as research funding application forms, that still use it. 

***Generalisation*.** In quantitative research refers to the transfer of results to the population from which the sample is taken, i.e. the part that has been taken (in some way), represents the whole. Here, generalisation is nomothetic. In qualitative research, this generalisation is about the case or phenomenon investigated; the findings of a given study can be used in other places or by different groups sharing similar experiences and/or conditions. Here it is an ideographic generalisation that can also be called *transferability* or case generalisation.^14^ For example, Charmaz, speaking of the experience of a woman with a chronic illness, points to the case by saying that “like many other chronically ill people”^15^ she resists describing herself in a way that might undermine her worth and elicit moral judgements. This does not mean that everyone with chronic conditions experiences what Cristina does, but rather that this experience is present in the phenomenon of chronicity. What is the use of this? The answer is simple: to be able to recognise it when it occurs.

***Design*.** As already mentioned, in quantitative research, the design is a set plan, whereas in qualitative research, it is a guide, with varying levels of flexibility, that the researcher develops throughout the study. Therefore, the study is not designed in a desk, but in the actual act of researching. *Data*. In quantitative research, data is already formed and available to be collected. In qualitative research, data is constructed, built during fieldwork, as the researcher interacts with study participants who narrate or describe their experiences. The data is subjective, not out there to be picked up. Consequently, the process of ‘collecting’ it will be different in qualitative research.

***Results*.** In quantitative studies, the results are a mathematical representation of the world and are separate from the analysis stage^6^. The results of a qualitative study are meaningful to others, credible, rich in detail and deep in meaning, with the potential to impact those concerned. ^16^ They are constructed as the data is analysed, so that the result is an evolution of the analysis. As there is no test in the form of a hypothesis, but rather a discovery, what qualitative studies produce are findings, although in some texts the term result might be used.

### 2.2. Same terminology but different ways of proceeding

The techniques for sampling, data collection, analysis, rigour and ethics share the same terminology in quantitative and qualitative methods, although, due to differences in paradigm, they are approached differently as the sequence in which they are carried out. Let us examine these procedures:

*Sampling.* In quantitative research, sampling is based on hypotheses and variables defined around the chosen sample (group, population). In qualitative research, it is done around the phenomenon or issue being researched and, at the same time, discovered. Therefore, it is not done all at once, but it will be sequential: cases and instances are selected as the study unfolds. Thus, sampling is not about participants themselves, but on their experiences or events that are revealed during the study. Furthermore, given that reality in qualitative research is believed to be constructed in action, sampling will seek informants who have first-hand experience of the phenomenon under study, i.e. who have constructed it. It is essential to be clear about this when designing the study and during fieldwork, otherwise, the researcher might get lost in the world of variables and questions that seek confirmation of theories or relationships, straying in this way, from the true meaning of qualitative research. If, for example, in a study, a person talks about the difficulties he has in understanding the instructions for taking medication and the researcher inquiries about his level of education and income, s/he will seek, in a positivist manner, causes for what is happening and not descriptions of how it happens, leaving aside issues that will reveal what is happening to that person.

***Data collection*.** In quantitative research, the researcher must remain as detached as possible from the data and not interfere in the data collection process. There should be no interaction with the study subjects, and the data must not be contaminated to remain objective. In qualitative research, on the other hand, the researcher establishes, during fieldwork, a close relationship with the people and their experiences to capture the subjectivity of the experience; being objective is not a goal since, as Leininger states, understanding one human being requires another human being.^17^ Qualitative data is not really collected but rather *generated* through the relationships that the researcher builds with the study participants. Therefore, in the context of qualitative research, we can talk about data collection, but we must understand that it implies something different from the quantitative context. Likewise, data generation is not guided by prior theories, but rather by what the analysis reveals. Therefore, it is not a separate stage as in the case of quantitative studies.

*Techniques for collecting/generating data*. Techniques for collecting quantitative data are structured and close, do not change throughout the study, and are aimed at capturing data objectively. In contrast, qualitative techniques are open and flexible. As the study unfolds, the techniques change and can be structured based on the analysis. The three main techniques for obtaining data in qualitative research are: interviews, observation, and document consultation. In quantitative studies, an interview consists of closed-ended questions, as the objective is to confirm or refute what is known or believed to be known. The interview contains many questions that correspond to variables or assumptions about what is being studied. Given that the essence of qualitative research is to discover, in the sense of revealing what is not obvious and naming it for the first time, the interview will have an exploratory objective, to delve into the unknown that is, a subjective experience; hence, it will be more of a conversation than an interrogation. So, a common way to begin a qualitative interview is to ask the participant to talk about her/ his experience (for example, as a patient, as a family member) and from here, as in a social conversation, the researcher will ask questions prepared in advance and address others that arise in the conversation, showing an interest in learning more about the experience. The questions will, therefore, be open-ended, few and worded as neutral as possible, i.e. without assumptions or preconceptions. 

Observation, as a technique for collecting data in quantitative research, is deductive. It records and counts what the researcher has decided in advance is interesting or necessary to count. The researcher's position, as in the interview, is external to what s/he is observing, without interacting with subjects, to ensure the objectivity of the data. Qualitative observation, on the other hand, is done to discover or reveal what is not seen or said, rather than to verify the frequency of pre-established categories. The researcher enters the field of observation as a theoretically sensitive explorer, that is, with some categories or ideas in mind to access the data. ^18^ This observation is participant, as data is obtained by participating in a scenario and sharing the experience with those who are there. During the observation, the researcher interacts with people and becomes part of the setting, rather than standing back and observing it from the outside.

By participating in the experience, the researcher will be very close to the insider's point of view and will be able to ask relevant questions to guide what to observe. For example, during fieldwork in which I accompanied community nurses on home visits in deprived areas of a city, I wondered how, in these noticeable conditions of scarce social and material resources, the nurses made relevant the message of prevention and promotion of child health. Specifically, before each visit, I wondered how they would gain access to the homes and win the trust of the people who lived there. The observation periods revealed that thy achieved that, by doing a work that they were not supposed to do, as it was not part of nursing care, and a work that was not expected of them going beyond their duties by for example, accompanying a mother of a baby to a hospital appointment. In doing his type of work, they responded to felt needs of the people they visit and expressed their commitment to their well-being. This was work on the margins of the professional duty, but necessary to do as it paved the way for doing the work they were entrusted with. ^19^

In this vein, the study of texts and documents in quantitative studies consists of inspecting documents to determine the frequency of certain chosen terms, and the text is treated objectively, as a reflection of reality. In qualitative research, writings are studied to identify the categories used in the document, describe them, and ultimately understand them. Texts are understood as products or artefacts created for a specific purpose. Regarding this, a well-known example is Durkheim's study of suicide. Based on mortality records showing fewer suicides among Catholics than among Protestants, he supposed that Protestants committed suicide more often. What was happening was that suicides among Catholics were not declared as such on medical certificates so that these people could be buried in the cemetery. Mortality records were socially shaped; they were not objective documents.

**Figure 1:**
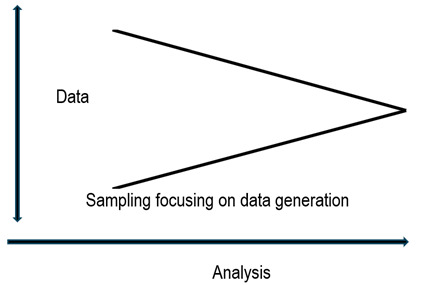
Concurrency of analysis and data generation

*Data analysis*. Quantitative analysis is statistical, and mathematics is the canon or ideal of the method. The way of analysing qualitative data, on the other hand, is derived from non-mathematical but interpretative procedures. In this analysis, concepts are constructed or categories are discovered through cognitive processes such as apprehension and synthesis. ^20^ In qualitative studies, data generation and analysis are done concurrently and guided by sampling. The concurrence is such that the first level of analysis occurs when the researcher interprets, from all the information generated, that which is relevant to the study.^21^ In short, data generation, sampling, and analysis go hand in hand, and this is what makes the study design evolve as it progresses, as illustrated in Figure 1.

*Rigour.* In quantitative studies, rigour relies largely on the objectivity and precision of the mathematical procedures used; study’s results must be valid, and the study must be replicable. On the other hand, the rigour of qualitative research lies in the researcher's approach, in how they carry out the research process, rather than in the techniques themselves. The rigour of a qualitative study is shown by its authenticity and credibility. ^22^

*Ethics.* In quantitative studies are embodied in the approval of the study by an ethics committee. In qualitative studies, although there is approval from an ethics committee, ethical issues will be present throughout the research process because, as the design is emergent, it is not possible to anticipate what will arise. This can be considered situational ethics, where the researcher considers the best way to proceed in each situation. ^23^. For example, Lipson narrates that during fieldwork she achieved such trusting relationships with study participants that they forgot she was a researcher and revealed information that could put them at risk. ^24^ In this situation, Lipson reminded them that they were providing information for a research study, at the risk of not generating data. Clearly, the researcher put the well-being of the informants before her study. As equality is a basic principle of qualitative research, it is also a relational ethic. As shown, the procedures are different in quantitative and qualitative research, as is their sequence. In quantitative research, the stages are separate from each other; one must be completed before the next can be addressed, and they follow an unalterable linear sequence. In qualitative research, the stages are concurrent and interact with each other, feeding off each other. Hence, rather than stages of research process, we can think of cycles in which converge data generation, analysis, rigour, ethics, and, in the last cycle, the writing of the findings.

## 3. Concluding remarks

**Figure 2:**
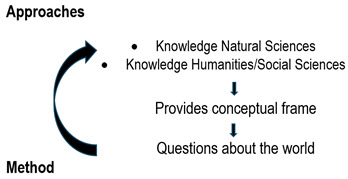
Approaches and conceptual framework

Given the characteristics of qualitative research and its differences from quantitative research, a researcher may ask him or herself, how do I choose between one method or the other? In their influential work on qualitative research in nursing, Field and Morse^25^ point out that the choice of research method is determined by three factors: a) the nature of the problem, i.e. the purpose of the study or what we want to know about a human issue; b) what is known about the subject to be investigated, both in quantitative and qualitative terms; and c) practical considerations, such as the experience of the researcher, the study participants, or the location where the study is to be conducted. While this reflection is valid in that it subordinates the method to the research question or the purpose of the study, let us not fool ourselves: asking questions in a qualitative manner is not a technical matter but a paradigmatic one. Without a paradigm shift from positivism, there can be no qualitative research. Figure 2 illustrates these ideas.

To produce scientific knowledge, we have two basic perspectives or approaches, one that comes from the natural sciences and the other from the humanities or social sciences. These approaches provide us with a way of seeing the world or conceptual frame. For example, in Clarke's lucid essay ^26^ on paradigmatic approaches in the sociology of medicine, she points out that, in an approach derived from functional structuralism, medicine is considered an institution of social control where the doctor designates, for example, an adequate patient’s behaviour or certifies the legality of his absence from work. In an approach derived from critical theory, on the other hand, medicine is seen as an ideology of social control where the healthcare system reflects socioeconomic, racial and sexual stratification and where technology, knowledge and medical skills are geared towards strengthening class relations. 

Finally, in a constructivist approach with an interactionist framework, medicine is considered an activity of social control in which its characteristics are described, and language is examined as symbols that have different meanings. These lenses or ways of conceptualising what is to be studied will point to the questions to be asked, which in turn will set in motion the method for answering them. Once the study is completed, it will feed into a type of knowledge: the nomothetic knowledge of quantitative studies or the ideographic knowledge of qualitative studies.

In summary, qualitative research has its own entity and, therefore, its own language and specific procedures that generate scientific knowledge, which cannot be produced by other means and is relevant in the field of health. The procedures in qualitative research are derived from a way of thinking and seeing the world that is acquired gradually through study and practice. To this end, I propose two exercises to reinforce what has been learned here.

## 4. Exercises

1-I suggest you identify the aptitudes you have for qualitative research work and what would make it difficult for you. 

Reflect: What can I do to improve it?

2-Answer the following questions:

- In what different ways is the researcher an instrument in a qualitative study?

- Why is qualitative sampling sequential?

- What type of generalisation is used in qualitative studies?

- Why is qualitative data obtained rather than collected?

- What is rigour in qualitative research related to?

Review your answers by rereading the article.

## References

[1] von Wright GH (1987). Explicación y Comprensión.

[2] Savin-Baden M, Major C (2025). Qualitative research: The essential guide to theory and practice.

[3] Denzin NK, Lincoln YS (2005). The Sage handbook of qualitative research.

[4] Wise BV (2002). In their own words: the lived experience of paediatric liver transplantation. Qualitative Health Research.

[5] de la Cuesta-Benjumea C (2006). "Aquí cuidamos todos": asuntos de individualidad versus colectividad en un estudio sobre cuidado en la casa de pacientes con demencia avanzada. Forum: Qualitative Social Research.

[6] Hesse-Biber SN, Leavy P (2011). The Practice of Qualitative Research.

[7] Eco U (2013). Kant y el ornitorrinco.

[8] Marshall C, Rossman GB, Blanco GL (2022). Designing Qualitative Research.

[9] Sudnow D (1971). La organización social de la muerte.

[10] de la Cuesta-Benjumea C (2011). La reflexividad: un asunto crítico en la investigación cualitativa. Enfermería Clínica.

[11] Martin K (1988). When a baby dies of SIDS. The parents’ grief and search for reason.

[12] Schatzman L, Strauss AL (1973). Field research. Strategies for natural sociology.

[13] Charmaz K (2014). Constructing Grounded Theory: A practical guide through qualitative analysis.

[14] Polit DF, Beck CT (2010). Generalization in quantitative and qualitative research: myths and strategies. International journal of nursing studies.

[15] Charmaz K (1999). Stories of Suffering: Subjective Tales and Research Narratives. Qualitative Health Res.

[16] Sandelowski M (2004). Using qualitative research. Qualitative Health Research.

[17] Leiniger M (2003). Asuntos críticos de investigación cualitativa.

[18] Blumer H (1967). Symbolic Interactionism.

[19] de la Cuesta-Benjumea C (1993). Fringe work: peripheral work in health visiting. Sociology of Health and Illness.

[20] Morse JM (2003). Asuntos críticos en los métodos de investigación cualitativa.

[21] Glaser B (1998). Doing Grounded Theory: Issues & Discussion.

[22] Hammersley M (2018). Routledge Revivals: What's Wrong with Ethnography? Methodological Explorations.

[23] Norris C (1993). Interpreting the field. Accounts of ethnography.

[24] Lipson J (2003). En: Morse JM, Asuntos críticos en los métodos de investigación cualitativa.

[25] Field PA, Morse JM (1985). Nursing Research: The application of qualitative approaches.

[26] Clarke J (1999). Salud y enfermedad-Lecturas básicas en sociología de la medicina.

